# Acceptance and Disclosure: Comparing genetic symmetry and genetic asymmetry in heterosexual couples between egg recipients and embryo recipients

**Published:** 2016-03-28

**Authors:** R Hertz, MK Nelson

**Affiliations:** Wellesley College, 106 Central Street, Department of Sociology, Wellesley, MA 02481, USA.; Middlebury College, Department of Sociology and Anthropology, Middlebury, VT 05753, USA.

**Keywords:** Disclosure, embryo donation, gamete donation, heterosexual women, married mothers

## Abstract

This paper explores the attitudes and experiences of 203 women in heterosexual couples who conceived via donated eggs (145) or donated embryos (58) in the last 5 years. Online surveys were sent to former patients (from many different countries) of a private Spanish clinic. By comparing the women in heterosexual couples who relied on donated eggs with those who relied on donated embryos, we examined the meaning of the absence of a genetic tie to a child in the two different situations - that of “genetic symmetry” where neither parent has a genetic tie to that of “genetic asymmetry” where only the mother does not have a genetic tie. No existing study has yet shown whether women who rely on donated eggs and women who rely on donated embryos have similar or different attitudes towards issues surrounding the experience of non-genetic motherhood. Three issues are discussed: (1) attitudes toward the importance of genetic ties and genetic information from the donor before and after the birth of their children, (2) patterns of disclosure, and (3) the relationship between attitudes toward genetic information and disclosure decisions. This study showed that although the two groups of women have many of the same attitudes, including attitudes toward the importance of genes as determinants of outcomes for the child, egg recipients are more likely than embryo recipients to agree that the genetic origins are important to them and that their children have a right to know genetic information. We also found that those who conceived with donated eggs more frequently disclose the nature of their conception to their child than do those who conceived with donated embryos.

## Introduction

For years most of the research on reliance on donor gametes has been about families constructed with the use of donated sperm. Not only does reliance on donated eggs or donated embryos create new scenarios, but the two cases are quite different. In the case of reliance on donated eggs, genetic asymmetry is altered within a two-parent, heterosexual family from what was the case when the family relied on sperm donation. In both circumstances of donated eggs and donated sperm, a woman carries and gives birth to a child and one parent still has no genetic tie to that child. In the case of donated eggs, it is the mother whereas in the case of donated sperm it is the father. Reliance on donated embryos introduces yet another variation. Genetic symmetry emerges in a new form within the two-parent, heterosexual family: while the woman still carries the child, rather than both parents having a genetic tie to the child (as would be the case in “normal” reproduction), neither parent has a genetic tie to the child. This paper considers several issues created within these two new scenarios.

Research shows that the desire to carry a child and to give birth to biological children of one’s own drives the search for fertility treatment rather than adoption (Hamilton et al., 2007; Park and Hill, 2014; Chateauneuf and Ouellette, 2015; Goldberg and Scheib, 2015). Less research has been directed at the degree to which donor eggs and donor embryos satisfy those concerns for women (Kirkman, 2008). Daniluk and Koert (2012) examined 2000 childless women and 599 childless men in Canada to assess attitudes toward the use of donor gametes. They found that the women were less willing to consider using donated eggs and embryos than were the men and that the reverse was true for donated sperm. They also found that although there was only a slight difference among the women in their attitudes toward donated eggs and donated embryos with more women expressing a willingness to use donated eggs than donated embryos, many more women were willing to use donated sperm than either donated eggs or donated embryos. A study among 140 cohabiting, childless women in Sweden found that only 25% would consider sperm donation but that even fewer (15%) would consider oocyte donation (Petersen et al., 2015). However, no existing study has yet shown whether women who have come to rely on donated eggs and donated embryos have similar or different attitudes towards issues surrounding the actual experience of non-genetic motherhood including assessments of the importance of genetic material.

One of the major topics in studies of reliance on donor gametes has been how families managed at the social-psychological level (Lycett et al., 2004; Bos and van Balen, 2008; Nordqvist 2012; Blake et al., 2014; Golombok et al., 2013). Some studies have compared parent-child relationships within different forms of assisted reproduction (donor insemination, IVF, egg donation and surrogacy) (Murray et al., 2006; Owen and Golombok, 2009) and between children conceived through assisted reproduction and children who were conceived “naturally” (Golombok et al., 2006). Within those broad topics, disclosure has been an issue of particular importance. Because of concerns about whether a donor-conceived child could cope with the knowledge of that conception, many people have argued against disclosure (Snowden and Mitchel, 1981; Shenfield and Steele, 1997; Walker and Broderick, 1999; Baccino et al., 2014). Now disclosure is often recommended by mental health personnel in the field of assisted reproduction because of the arguments of some that children will be harmed by not knowing the truth about their genetic origins (Human Fertilisation and Embryology Authority, 2007; Readings et al., 2011). Even so, disclosure is still not invariably the case. Intention to disclose and the effect of disclosure continue to be important topics of investigation. Most of these studies have looked only at the use of donor sperm, although some have examined the intentions and likelihood of disclosure in cases of donor eggs (Murray and Golombok, 2003). Other studies have also examined that issue comparing reliance on donor sperm and donor eggs (Landau et al., 2008; Readings et al., 2011).

Even though there is little research on couples who have conceived through donor embryos and their intentions to disclose, Söderström-Anttila et al. (2001) reported that while in theory most of the 27 couples they surveyed in Finland about embryo donor conception thought that children should be told about their conception, among the couples who had children at the time of their study, only a few had disclosed. Through semi-structured interviews, MacCallum and Golombok (2007) examined disclosure patterns of 21 UK heterosexual couples with children between 2 and 5 years old who conceived via embryo donation. At the time of their research they found that only 2 mothers had told their child about the method of conception and another 5 said they were planning to disclose in the future. The majority was inclined toward nondisclosure, although 15 respondents had disclosed to at least one grandparent. Following up on a sample of embryo donation mothers, MacCullum and Keeley (2012) found that the 17 embryo donation mothers were much less likely to disclose the method of conception to their children than the 24 mothers with an adopted child and the 28 mothers who had genetically related children conceived with the use of IVF. They argued that legally, practically and psychosocially there is a vast difference between adoptive children and children conceived with donated gametes. Furthermore, in some studies where parents relied upon donated gametes, parents engaged in “partial disclosure” where they might tell the child about IVF use but not gamete use (Readings et al., 2011) or the use of a sperm donor but not an egg donor in the case of single mothers (Landeau et al., 2008). In these studies parents are not disclosing their own lack of genetic relatedness to the child.

To date, we only found one study that has compared the likelihood of disclosure between embryo donation, sperm donation and egg donation (Baccino et al., 2014). That study examined three different types of families (heterosexual families, lesbian couples and single mothers). They found no difference between type of treatment and intention to disclose within heterosexual families. The authors conducted no follow-up study to show whether parents actually did what was intended. Indeed, no study has directly compared decisions about disclosure among mothers who were egg recipients and mothers who were embryo recipients. This study begins by addressing that issue among a sample of women in heterosexual couples, some of whom have relied on donated eggs (using their partner’s sperm for conception) and others who have relied on donated embryos. We place decisions about disclosure in the context of other parental attitudes about the importance of having genetic information.

Studies of disclosure and parental attitudes toward the donor have become entwined with studies about donor anonymity, especially in those countries where legislation allows offspring and their parents to have access to information about the donor. This is not the case in Spain, where this study was conducted. Spanish law still protects the lifelong anonymity of all medical donors (Baccino et al., 2014). Of course, not all patients in Spanish clinics are themselves citizens of Spain. Spain is an important destination for cross-border reproduction because it has limited restrictions, short waiting lists and enough available gamete donors (Shenfield et al., 2010; Culley et al., 2013; Gomez and de La Rochebrochard, 2013; Krolokke, 2014).

In short, this paper looks at women in heterosexual couples in a Spanish clinic (some of whom crossed borders to be treated there and some of whom did not). It compares mothers in situations where they experience genetic symmetry with their partners (reliance on embryos) as opposed to those situations where they experience genetic asymmetry with respect to three issues: (1) attitudes toward the importance of genetic ties and genetic information from the donor before and after the birth of their children, (2) patterns of disclosure and (3) the relationship between attitudes toward genetic information and disclosure decisions.

## Methods and Materials

### Data Collection

The data came from an online survey of former patients who were successfully treated by a fertility clinic in Spain and who came from many countries. Clinic personnel sent an email invitation to all former patients (1296) who conceived and gave birth to children through donor gametes or embryos in the last 5 years. The email offered links to the survey with four language options: English, Spanish, French and German. The survey options were translated and put online by American translators. To ensure that the survey translations, especially the technical language of gametes, were accurate the clinic’s personnel commented on each translation. The survey was online from November 15, 2015 to January 15, 2015. Ethical approval for this study was obtained from the Institutional Review Boards of the US institutions represented by the two authors. The overall response rate was 23%.

### Measures

The survey collected background information about the respondents including, for those who had crossed a border to be treated in Spain, the reasons for doing so; information concerning the experience of, and attitudes toward reliance on donated gametes; and general attitudinal questions about a range of issues including the importance of genes and government regulation of fertility treatment. The majority of the survey consisted of closedanswer responses. One open-ended question, at the end of the survey, asked respondents to comment on what they had learned through their experience of conception with gamete donation. All questions were pretested. Some of the questions had previously been used in earlier studies of US donor-conceived families (Freeman et al., 2009; Sawyer et al., 2013; Hertz et al., 2016).

Most attitudinal questions under consideration were scored on a five point Likert scale from “strongly agree” to “strongly disagree.” For these items we used “strongly agree” (and in two cases “strongly disagree”) as opposed to means because means make assumption about the scale data. By using “strongly agree” or “strongly disagree” we do not assume the ordinal measure is a continuous scale. “Strongly agree” and “strongly disagree” respondents held unambiguous attitudes. The one exception to this practice is the set of responses to a question that asked how important respondents thought genes were as determinants of a series of outcomes. Responses were scored from 1 (“very important”) to 4 (“not at all important”). We report means (and standard deviations) for these data because the scholars from whom this question was adopted relied on that form of analysis (Shostak et al., 2009).

### Data Analysis

Data analysis was conducted with the use of SPSS. Tests of significance are reported as they are appropriate given the form of data. We asked one open-ended question. Codes for the single openended question were developed by the first two authors using a traditional grounded theory approach (Glaser and Strauss, 2009). This question was coded separately by the two authors to insure inter-coder reliability. When there was disagreement about the coding, the responses were coded as “other”. No respondent is quoted more than one time.

### Participants

Since we are especially interested in issues of genetic symmetry and genetic asymmetry within a family, we included only those survey respondents who were women in a heterosexual partnership at the time they relied on either donated eggs or donated embryos. Because only four men whose wives had conceived via their own sperm and donated eggs answered the survey, a comparable analysis could not be conducted among the men.

A total of 203 out of 297 respondents met the criteria for inclusion (68%). Among the women, 145 (71%) had relied on egg donation and 58 (29%) on embryo donation. Those who responded to the online survey are representative of the population treated at that time by this clinic. This means that the percentages of respondents who conceived via egg donation and those who conceived via embryo donation are similar to the proportions treated with the use of these types of donations within the clinic population.

As shown in Table I, the women receiving donated eggs and those receiving donated embryos were the same age when they answered the survey. They also had similar incomes, employment status, locations and total number of children. The majority of women in both groups were Caucasian. The women in the two groups did come from different countries. Almost half of the women seeking donated eggs came from England in comparison with only a quarter of those seeking donated embryos. Moreover, the two populations of women who were not Spanish citizens also crossed borders for different reasons. More women who received donated embryos than those who received donated eggs indicated that they came to Spain because the treatments performed in their home country were not successful.

**Table I:** — Demographic data and reasons for border crossing.

**IA. Demographic Data**		**Egg Donation** **(N = 145)**	**Embryo Donation** **(N = 58)**	**Chi-square test** ***p-value***
Mean age (Standard Deviation)		45.4 (4.86)	45.3 (4.24)	
Mean Number of children (total)		1.9 (0.89)	1.8 (0.91)	
Percent living in City or Suburbs		43	43	
Percent who make Less Than 80,000 €/year		44	53	
**Type of Employment (%)**		45.4 (4.86)	45.3 (4.24)	
	Homemaker	25	25	
	Full time employed	33	40	
	Part time employed	38	35	
	Unemployed	4	0	
	Total percent	100	100	
**Main Citizenship (%)**				
	Canada	1	0	
	England	48	24	
	France	3	2	
	Germany	8	9	
	Italy	1	2	
	Ireland	8	14	
	Netherlands	1	3	
	Northern Ireland	2	3	
	Scotland	6	7	
	Spain	15	24	
	United States	1	3	
	Australia	6	5	
	Sweden	1	0	
	Norway	2	3	
	Wales	1	0	
	Total Percent	100	100	
**IB. Reasons for Border Crossing (%)**		**Egg Donation** **(N = 124)**	**Embryo Donation** **(N = 44)**	
Home country wouldn’t allow services		9	16	
Not enough donors in the home country		48	39	
Wanted anonymous donor		36	32	
Wanted gamete not allowed in home country		15	21	
Shorter wait times than in home country		58	48	
Tried treatments in home country that did not work		**32**	**64**	***p*-value = 0.001**
Partner tried treatments that did not work		2	7	
Procedures cost less than in home country		7	16	
Costs for gametes less than in home country		1	7	

## Results

### The Importance of Genes and Genetic Links

None of the women in this study had a genetic (DNA) link to her child. As Table II shows, almost a third of the women in each group indicated that prior to the birth of her child they had found this absence painful. In response to a question at the end of the survey asking what advice respondents would have for individuals considering gamete donation, a woman who conceived via a donated egg and her partner’s sperm said, *“Coming to terms with your own genetic loss will help you help your child when they realize what the loss means to them. You can’t have the gain without the loss and mine is a glass half full of joy!”*. A woman who conceived via a donated embryo gave a very similar response: *“If there is no other way this is the way! Be ready to get used to the fact that the child will just not be connected genetically with you”*. One mother who had a child conceived with a donated egg and her partner’s sperm expressed gratitude toward the donor, delight in seeing that her children share their father’s features, and poignant acceptance about seeing the donor reflected in her children: *“My children were twins. They looked very alike. Like each other and their father and father’s family. Luckily also like their naturally conceived big sister. But they share features of their donor mother also. I can see a little of her face and hands in each of them and thank her in my heart each time I do”*.

Even though the absence of a DNA tie was initially painful for some women in each group, very few (4%) of the women in both groups of mothers responded that prior to giving birth they had worried at all that they would not bond with the child and almost half the women in both groups strongly disagreed with a statement to that effect (Table II). More than half of the women who conceived via donated eggs and their partner’s sperm said that they were glad that their partner would have a genetic tie to the baby because that was important to themselves or their partner. One woman, who provided a more fully developed response than other respondents, situated her thoughts this way when asked at the end of the questionnaire what advice she had for others:

**Table II:** — Attitudes towards genetic ties and motherhood (NA = not applicable).

**BEFORE BIRTH**	**Egg Donation** **(N = 145)**	**Embryo Donation** **(N = 58)**	**Chi-square test** ***p-value***
	**% who strongly agree**	**% who strongly agree**	
It was initially painful to think this child would not be genetically related to me	28	30	
I did not realize how much my feelings about being a woman were tied to my ability to have a child that grew inside my body and I gave birth to	28	28	
My partner would have a genetic (DNA) link which was important to me	54	NA	
My partner would have a genetic (DNA) link which was important to my partner	53	NA	
It was initially painful to think this child would not be genetically related to my partner	NA	9	
I felt concerned because I knew having a genetic (DNA) link was important to my partner	NA	11	
**BEFORE BIRTH**	**% who strongly disagree**	**% who strongly disagree**	
I worried that I would not bond with this child	**60**	**43**	***p*-value = 0.03**
**AFTER BIRTH**	**% who strongly agree**	**% who strongly agree**	
I am glad to be a mom	97	97	
I think how a child is raised is more important than where their genes come from	80	78	
I still wish I had a genetic (DNA) link to my child	11	5	
We are an ordinary family	72	74	
My partner is too busy with our child(ren) to think about their origins	39	37	
I am too busy with my child(ren) to think about their origins	40	37	
My partner has a bond with this child	92	97	
I have a bond with this childm	94	97	
I think we are a closer family than others we see regularly.	14	21	
I feel I love my child(ren) just as much as other families love their child(ren).	66	60	

“Can’t comment, everyone is different. But I am very blessed to have my lovely twins and have never ever felt I love them less for not being mine genetically. I suppose I would have been interested to see my genetic children and note traits, but it would have only been for reasons of curiosity, and in fact my family have some health failings that I am pleased not to have to worry about passing on. That said, I think my husband is pleased they carry his genetics. I never doubted it, but my learning is that you will love your donor egg children just as much as if they came from your own eggs!!”

The respondents who relied on donated embryos did not express concern that their partners would not have a DNA link to the child: fewer than ten percent strongly agreed with the statement that the absence of a genetic link was important to them; about the same number of respondents strongly agreed with the statement that the absence of a DNA link was important to their partner.

However, the two groups of women reported having had at least one different feeling prior to giving birth. Fewer of the women relying on donated eggs strongly disagreed with the statement that they worried that their partner would not bond with the child, suggesting that in the situation where the father would not have a genetic tie to the child the parent worried more about the father not bonding.

After birth, the two groups of women did not differ significantly in their reported wish to still have a DNA connection to their child. Relatively equal proportions of the women in each group believed their family to be closer than other families, believed their children were the same as other children and believed they loved their children as much as other people loved theirs. Moreover, the vast majority of women in both groups said they were glad to be a mom, they believed that how their children were raised was more important than genetic influences, they now felt like an “ordinary” family, and both they and their partners had bonded with the child.

These attitudes both of delight in having a child and of viewing the absence of a genetic link as being of little significance are apparent in the responses to the open-ended question at the end of the survey. Among the egg recipients who responded, twothirds (67%) were positive about the nature of their conception. Among those, twice as many mentioned that their joy in parenthood came in spite of the absence of a genetic connection in comparison with those who simply spoke of the joy of parenthood without mentioning the genetic issue. Two women who conceived via donated eggs illustrate this frequent response: *“It doesn’t matter that your child was not conceived using your eggs. The love you have for them is the same as if they were. I love my daughter more than anyone I have ever loved before and I can’t imagine that love being any stronger if she had been conceived with my eggs. She’s the most amazing thing that has ever happened to me”* and *“You forget that you used a donor the moment you are pregnant. It feels natural and the bonding is instant. The level of happiness you feel is not able to be measured. My husband was so grateful I talked him into it”*.

These same attitudes are also apparent in the comments written by women who conceived via donated embryos. The overwhelming majority of these comments (88%) were also positive and, once again, the insignificance of genetic ties was mentioned twice as often as it was ignored, as these two comments illustrate: *“I love my child as if the child were genetically linked to us – genes are not the most important issue when raising a child – love is the most important thing”* and *“I would suggest the parents try not to worry, I worried about all sorts of things, but in reality ‘real life’ takes over. The main thing is to be parents. When your son is in your arms you forget whether it’s yours or not genetically”*.

As much as intimacy bound parents to children, women often commented that they felt grateful for the donor’s generosity (Kirkman, 2008). A woman who conceived with donated egg and her partner’s sperm expressed herself this way: *“Thanks to a donor that I could be a mother and I can never thank that anonymous person and tell her how happy she made me and I am. Thank you”*.

One woman who conceived via embryo donation similarly expressed her gratitude as follows:

*“It has been a wonderfully positive experience for me and I am grateful that I could avail of double donation in order to have a child. I feel very proud of my son and extremely grateful to his donors. I frequently think of them in an abstract way and feel very lucky”*.

Respondents were asked how important they thought genes were as determinants of a series of outcomes on a scale from 1 (“very Important”) to 4 (“not at all important”). As shown in Table IIIA, the two groups of women have the same attitudes towards the importance of genes as determinants of the outcomes for a child. In this context, where the two groups of respondents held similar attitudes about the significance of genes for shaping outcomes, it is especially interesting to note that those who received donated eggs more often strongly agree with two statements: “children have a right to know their genetic origins” and “it is important to me that my children know their genetic origins” (Table IIIB). There were no statistically significant differences between the two groups in the proportion saying that parents should not have “secrets” about their child’s conception or in the proportion making other comments about the importance of the donor and the information the donor could provide. However, in each of those cases, more of the egg recipients than embryo recipients strongly agreed with the statements. One donated egg recipient reflected on how the issue of the source of her child’s genes increasingly bothered her over time, especially when health issues (which were only reported by a minority of respondents) emerged in her children: *“Don’t underestimate the change of heart you may experience about the genetic question once your child is born. Now my boys have had a couple of health issues I realize I didn’t know enough about the donor to make an informed decision”*. Another egg recipient, in contrast with the one just quoted, noted that further knowledge about her child’s genetic background could simply satisfy her curiosity and eventually, that of her child: *“I love my little child very, very, very much. But sometimes I am so curious about her biological [genetic] parents, and for her later”*.

**Table III:** — Attitudes toward genetic information.

**IIIA: Degree to which respondents believe differences in in genes are responsible for:** (means [standard deviations])		**Egg Donation** **(N = 145)**	**Embryo Donation** **(N = 58)**	**Chi-square test** ***p-value***
The major illnesses they will develop in life		1.72 [0.69]	1.66 [0.58]	
Whether or not they will develop a serious mental illness		1.86 [0.68]	1.86 [0.67]	
Their overall personality		2.29 [0.82]	2.23 [0.71]	
Their general intelligence		2.09 [0.77]	2.07 [0.67]	
Their success in life		2.85 [0.76]	2.93 [0.75]	
Their level of math ability		2.27 [0.75]	2.21 [0.72]	
Their level of athletic ability		2.23 [0.74]	2.18 [0.73]	
Their level of artistic ability		2.28 [0.79]	2.17 [0.68]	
**IIIB: ATTITUDES TOWARD HAVING GENETIC INFORMATION** (Percent who “strongly agree”)		**Egg donation**	**Embryo donation**	
Children have a right to know about their genetic origins if they want that		**24**	**10**	***p*-value = 0.02**
Families should not have “secrets” about genetic issues		33	28	
It is important to me that my children know their genetic origins		**19**	**7**	***p*-value = 0.04**
Knowing the donor could make my life easier 0 2		0	2	
Donors could offer my child more insight into the origins of genetic traits		10	7	
Donors could offer my child more insight into the origins of personality traits		8	5	
**IIIC: PERCENT WHO DISCLOSED WITHIN ATTITUDES TOWARD HAVING GENETIC INFORMATION** (Within Categories of “Strongly Agree” and Conception Method)		**Egg donation**	**Embryo donation**	
**It is important to me that my children know their genetic origins *(showing % and number on which % is based)***				
	Agree Strongly	**48% (25)**	**8% (13)**	***p*-value = 0.01**
	Agree, Neutral, Disagree, Disagree Strongly	**21% (120)**	**11% (45)**	
	**Chi-square test p-value**	***p*-value = 0.00**		
**Children Have a right to Know their genetic origins if they want that information *(showing % and number on which % is based)***		**Egg donation**	**Embryo donation**	
	Agree strongly	46% (35)	17% (6)	
	Agree, Neutral, Disagree, Disagree Strongly	19% (110)	10% (52)	
	**Chi-square test p-value**	***p*-value = 0.00**		

### Attitudes towards and decisions about disclosure

As Table IV shows, egg recipients were more than two times as likely to disclose the nature of their conception to their oldest child conceived with donor gametes than were embryo recipients. This difference was not just the result of a difference in age of children among those relying on eggs and those relying on embryos. Among those with children under age 4, 19% of those conceiving with donated eggs had disclosed to their children in contrast with 10% of those conceiving with donated embryos. On the other hand, among those with children over the age of 4, 36% of the former had disclosed in contrast with 15% of the latter.

**Table IV:** — Issues of disclosure.

	**Egg Donation** **(N = 145)**	**Embryo Donation** **(N = 58)**	**Chi-square test** ***p-value***
**Percent who Disclosed Donor Conception**	**26**	**10**	***p*-value = 0.02**
**Reasons for not Disclosing:** **(% each was a reason)**	**Egg Donation** **(N = 108)**	**Embryo Donation** **(N = 52)**	
Child is now too young and will tell when child is older	53	52	
Child is too young and might disclose when child is older	**43**	**28**	***p*-value = 0.04**
No reason to tell	26	15	
Don’t want child to feel different from other children	32	27	
I fear I won’t be seen as the real parent	39	40	
It would be too painful	**5**	**14**	***p*-value = 0.05**
**Most important reason for Not Disclosing:** **(% indicating this was the most important reason)**	**Egg Donation** **(N = 108)**	**Embryo Donation** **(N = 52)**	
Child is too young and might disclose when child is older	**14**	**2**	***p*-value = 0.02**
**Patterns of Discussion and Thought:**	**Egg Donation**	**Embryo Donation**	
Percent who never talk to partner about the donor conception	**31**	**47**	***p*-value = 0.03**
Percent who say they think about the egg donor less often than once a year	**27**	**48**	***p*-value = 0.01**
**Who knows child conceived with donated gametes:** **(% indicating each person)**	**Egg Donation** **(N = 145)**	**Embryo Donation** **(N = 58)**	
No one	6	10	
My parents	57	45	
My partner’s parents	42	35	
My siblings	50	43	
My partner’s siblings	37	26	
Close friends	59	59	
Pretty much everyone we know	8	14	

Among those who had not disclosed, egg recipients were more likely than embryo recipients to say that they had not yet decided whether or not they would tell the child at a later time. A mother who conceived via donated eggs and her partner’s sperm, when asked what advice she had for other women relying on assisted reproduction technologies, expressed her concern about the issue of the genetic parentage of her child and disclosure: *“A bond to a child is not based on genetics. It is something that needs effort and is built slowly, just like any relationship in your life. If you accept this, then don’t hesitate. I don’t know how my children will respond to the knowledge that I am not their genetic mother. But I want to tell them when they are still young and I hope they will keep loving me the way they do now. And I hope they won’t find it distressing that they will never know who their genetic mother is”*.

By way of contrast, more of the embryo recipients said that they had not disclosed because it would be too painful to do so. An embryo recipient explained that she worried that telling her children would cause unnecessary anxiety: *“It is a huge decision with implications that you may not have thought about beforehand and emotionally can be uncertain at times as we love our children so much and want to do 100% right by them but protect them also. It can be a burden to carry at times re the donation but I think our children are very happy and giving them this info will be of no help to them and may make them become unsure of who they are. We do not want that to happen”*.

Other behaviors related to disclosure were consistent with the differences between the two groups in patterns of disclosure to children. Embryo recipients were more likely than the other women to say that they never talked with a partner about the egg donor (Table IV). Egg recipients were more likely to say that they thought about the egg donor more often than once a year. The mother of a child conceived with an egg donation reflected that thinking about the donor occasionally did not take away from her happiness: *“It may be that you don’t forget that there was a donor but it won’t stop [the children] being wonderful and a great joy”*.

Parents who have conceived via a donated egg or donated embryo also have to decide whether they will tell other people outside the immediate family about the donor conception. Given what has already been reported, it is not surprising to find that more of those who received donated eggs than those who received donated embryos disclosed to their relatives and close friends, although the differences were not statistically significant.

### The Relationship between Disclosure and Attitudes toward Genetic Information

As Table IIIC shows, the differences in rates of disclosure between egg recipients and embryo recipients remain in the same direction even when we examine those patterns within different responses to the questions concerning attitudes about the importance of genetic information for children and parents. Recipients of donated eggs were more likely to have disclosed than recipients of donated embryos, although in only one case did these numbers rise to the level of statistical significance. Attitudes were also important: among those who received donated eggs, those who agreed more strongly with the two statements about the importance of genetic information (to the parents and to the children) were more likely to disclose than those who did not agree strongly. This means that both attitudes and type of donor conception have an impact on rates of disclosure among those with donated eggs and, less strongly, among those with donated embryos.

## Discussion

### Limitations

These data came from an online survey distributed to former patients of a single clinic in Spain. Web surveys generally have relatively low response rates (Couper, 2000; Monroe and Adams, 2012) and this was also true for this survey. On the other hand, concerns about response rates have to be weighed against the advantages online surveys have for trying to reach a generally hard to reach population such as gamete recipients (Freeman et al., 2009). Our findings were also based only on women in heterosexual partnerships. These factors limit the generalizability of our findings. Patients came to the clinic both as citizens of Spain and as border crossers in search of reproductive care they could not get elsewhere. They therefore represent a number of different countries and their attitudes might be shaped by their nationality as much as by their method of conception (Voss, 2000; Purewal and van den Akker, 2006, 2007; Schnittker, 2015). Moreover, there were differences in nationality between the two groups of respondents – those who received donated eggs and relied on their partner’s sperm and those who received donated embryos. More of the women seeking donated eggs came from England where anonymity is not allowed than those seeking donated embryos. Given the particular set of regulations that prevail in Spain, where donor anonymity is the law, when respondents were asked questions about knowledge of genetic origins and knowing the donor, they responded about what they might have wanted, not what was actually possible for them in Spain. Finally, more of those who received embryos came to Spain because they had tried treatments that did not work in their own country, suggesting that the situation for embryo recipients was somewhat more urgent than for egg recipients. Any comparisons between the two groups of respondents should be read with these issues in mind.

### Attitudinal Differences

The two groups of women appear to have anticipated motherhood in similar ways. Almost a third of the respondents mourned the loss of a genetic tie while being secure that they would bond with the child (Ehrensaft, 2005). The women who did not use their partner’s sperm were more likely to be anxious about whether or not their partners would bond with the children. The women who did use their partner’s sperm took pleasure in the genetic link between their husbands and the children, both for their sake and for the sake of their husbands, while the vast majority of embryo recipients indicated that they were not concerned about the absence of a genetic link between child and partners. After birth the two groups of women responded similarly to a broad range of questions about satisfaction of being a parent and about both their own and their partner’s bond with the child. Our data suggest that when it is possible, a DNA link is valued. When this is not possible, respondents seem to adjust to the absence of that link and seem to believe that their husbands have also adjusted to the absence of that link. The positive comments we quoted above about relationships with non-genetic children illustrate these findings. Parents feel “blessed”; they say that they feel “no differently” about those conceived with their own gametes and those conceived with donated gametes.

Our two groups of women differed in their attitudes towards the overall significance of knowing about genetic origins with those who relied on donor eggs alone feeling more strongly that this knowledge is important (Strathern, 1999; Edwards, 2000; Almack, 2006; Thompson, 2007). Given this difference it is not surprising to find that disclosure to a child was more likely among egg recipients than among embryo recipients. This is contrary to the findings published by Baccino et al., (2014). Although the two groups of women were equally likely to plan to tell their children about their donor conception when the children were older, among egg recipients, non-disclosure seems to be more matter of fact: they saw no reason to tell their children about their donor conception. Among embryo recipients, non-disclosure seems to be due to both anxiety and fear (Kirkman, 2008). One woman mentioned that she thought information about the embryo donation could make her children “unsure of who they are”. This response is particularly interesting because in the situation of genetic asymmetry one might think that the women receiving eggs would be more worried about issues of parentage. Apparently, having one genetic parent in the family reduces that concern. Women who relied on donated eggs appear to feel more confident about the integrity of the family and the child’s response to disclosure because the child has a genetic link to one parent.

Although the two groups were equally likely to say that they would disclose to their children at a later date, other evidence suggested that disclosure of conception with donated gametes is a far more complex issue for the women relying on donated embryos. Almost half of those relying on donor embryos never discussed the donor conceptions with their partner. Our findings suggest that those who have conceived via donated embryos are more private about donor use than those who conceived with donated eggs and their partner’s sperm. This finding is similar to that of MacCallum and Golombok (2007).

The two groups of respondents appear to view their contribution to creating a child differently, even though in both cases the women were the gestational mothers. It may be that within a couple where the mother conceived with donated eggs, the ability to carry a child is more central to the production of their child than her genes. The couple converts her bodily contribution to the child as an equitable contribution, recreating a form of “symmetry”. Women who conceive with the use of donated embryos also give birth but there is no family lineage from the husband. It may be that this lack of a genetic link from both the maternal and the paternal side makes it more difficult to figure out how to inform the child. The lack of disclosure by mothers conceiving with donated embryos is in line with earlier research on disclosure in the case of non-genetic parenthood (Söderström-Anttila et al., 2001; Baccino et al., 2014). In those situations where there is no genetic link to the child, disclosure continues to be a troublesome issue. Further research should be conducted on these issues among a broader range of mothers (e.g., single women, women in lesbian partnerships) as well as among men who have children conceived with donor sperm, donor eggs and donor embryos.
